# Smoking Patterns and Anxiety Factors Among Women Expressing Perinatal Depression

**DOI:** 10.1089/whr.2021.0111

**Published:** 2022-02-10

**Authors:** Sotiria V. Anastasopoulou, Konstantinos S. Bonotis, Chrissi Hatzoglou, Konstantinos C. Dafopoulos, Konstantinos I. Gourgoulianis

**Affiliations:** ^1^ Department of Respiratory Medicine, Faculty of Medicine, University of Thessaly, Biopolis, Larissa, Greece.; ^2^ Department of Psychiatry, Faculty of Medicine, University of Thessaly, Biopolis, Larissa, Greece.; ^3^ Department of Physiology, Faculty of Medicine, University of Thessaly, Biopolis, Larissa, Greece.; ^4^ Department of Obstetrics and Gynecology, Faculty of Medicine, University of Thessaly, Biopolis, Larissa, Greece.

**Keywords:** smoking, perinatal depression, pregnancy, smoking cessation

## Abstract

***Background:*** Relationships among perinatal depression occurring a number of weeks before and after childbirth and smoking have been identified. Depression may lead to the inability to abstain from smoking during pregnancy.

***Objectives:*** This study aims to determine factors affecting smoking during pregnancy revealing potential relationships between depression and smoking patterns during and after pregnancy.

***Methods:*** A total of 206 mothers participated in the study. Data were collected through self-reporting as respondents were asked to answer questionnaires during the 12th week of pregnancy, during the 30th week of pregnancy, after childbirth, and during the period after pregnancy. Relationships between smoking behavior, sociodemographic variables, and feelings of perinatal depression were examined using chi-square test and binary logistic regression analysis. A follow-up investigation has been conducted after 2 years revealing the percentage of women returning to their smoking habits.

***Results:*** Smokers before (*B* = 0.568; *p* = 0.026) and during pregnancy (*B* = 1.238; *p* = 0.009) were more likely to express depression before childbirth. Average daily cigarette consumption before (*B* = 1.110; *p* = 0.001) and during pregnancy (*B* = 1.167; *p* = 0.002) was associated with depression during pregnancy. Women who smoked during pregnancy reported significantly more depressive symptoms after pregnancy (*B* = 1.757; *p* = 0.005) compared with nonsmokers and smokers who abstained during pregnancy. Average daily cigarette consumption during pregnancy (*B* = 1.402; *p* = 0.002) affects the expression of depression after pregnancy. Women who smoked before pregnancy (*B* = 0.568; *p* = 0.025) and their average daily cigarette consumption before pregnancy (*B* = 1.465; *p* = 0.025) were highly associated with the inability to abstain from smoking during pregnancy. However, the knowledge of risks of maternal smoking during pregnancy (*B* = −1.110; *p* = 0.001) and medical consult on abstaining (*B* = −1.238; *p* = 0.009) reinforced the maternal attempt to quit smoking. The follow-up investigation revealed an elevated amount of women returning to previous smoking patterns.

***Discussion:*** Perinatal depression is associated with smoking patterns during pregnancy. Assessment of depression and smoking is needed throughout perinatal period to support the health of women.

## Introduction

Smoking has been identified as one of the most harmful human habits, and in the prior decades, a significant increase has been observed in female smokers. In addition, depression is recognized as a major risk for the public health and is most prevalent among women.^1^ Studies have shown that one of five women will report at least one depression episode in their lifetime.^2^ Perinatal period, the period relating to the time, usually a number of weeks, immediately before and after childbirth, is considered the most frequent period in a woman's lifetime for the expression of depressive symptoms.^3^

Smoking during pregnancy causes obstetric complications, including miscarriage, placental abruption, ectopic pregnancy, premature rupture of membranes, preterm birth, low birth weight, and fetal death.^4,5^

*Perinatal depression* is defined as an emotional disorder that occurs before childbirth (*antenatal depression*) or even weeks after pregnancy (*postnatal depression*). Antenatal depression affects a large percentage of female population and seems to be associated with a significant risk for preterm birth,^6^ preeclampsia, and poor pregnancy outcomes.^7^ Pregnancy can be responsible for loss of energy and major changes in appetite and sleep, but these can also be signs of depression.^8^ Furthermore, motherhood and role changes can also affect the mental health of new mothers causing postnatal depression.^9,10^ During the period before and after childbirth, women experience huge hormonal fluctuations, which can lead to a high risk of expressing mood disorders and depression.^7,8^

Often women turn to smoking or consuming alcohol to relax and deal with the new demanding circumstances that prevailed in their lives.^11^ Their main goal was to improve their sense of well-being or even as a quick reward.^12,13^ Thus, reducing or quitting smoking can cause a reduction of depressive symptoms.^13^ Furthermore, the majority of women seem reluctant to abstain from smoking permanently after childbirth. Their emotional vulnerability poses a huge burden upon them, as new responsibilities arise disturbing their former everyday routine. Returning to their smoking habits, even shortly after labor, is considered as an attempt to regain their former way of living.^14^

## Objectives

Despite the extensive public health messaging of the hazards of smoking, particularly during pregnancy and while breastfeeding, many women keep on smoking. Therefore it is important to examine the reasons that provoke women to continue smoking and the emotional effects of their actions.

It is of high importance to define the percentage of women who deny abstaining from smoking during pregnancy and breastfeeding in association with the outbreak of perinatal depression as well as the percentage of those who manage to quit smoking.

The aim of this study was to obtain insight into the associations between smoking patterns and depressive feelings during pregnancy and after childbirth, taking into account the emotional changes and the anxiety factors that impose on women during that period.

## Materials and Methods

### Study design—sampling

The study design was observational and prospective. Pregnant women who had antenatal care and delivered at the University Hospital of Larissa from June 2012 to August 2014 and also at two private hospitals of Larissa from December 2012 to April 2016 participated in the study. Participants were included if they were at least 18 years old and had an adequate knowledge of Greek to answer the questionnaires. In an attempt to detect the initiation of depressive symptoms strictly during the perinatal period, thus a number of weeks immediately before and after childbirth, women who experienced depressive symptoms even before conception were excluded from the study.

A total amount of 206 women volunteered to participate. Written informed consent was obtained from respondents, all of whom were assured of confidentiality. Ethics approval was received from the Ethical Committee of the University Hospital of Larissa. The follow-up investigation has been conducted from December 2018 to September 2020, by personal contacting the participants of this study. Clinical Trial Registration number: 2586/18-01-2016.

### Measurements

Perinatal information was collected by means of questionnaires that comprised questions reflecting the life status of women before and during their pregnancy to depict their everyday life routine. In addition, the type of conception and the time interval until conception was reported. Respondents were asked to provide information about their previous and current smoking status as well as their daily cigarette consumption before and during pregnancy. Data were collected at four points in time: during the 12th week of pregnancy, during the 30th week of pregnancy, after childbirth, and during the period after pregnancy (about 2 months after childbirth). Additional data were collected within an average of 2 years after labor.

### Depression

To measure depressive symptomatology the Edinburgh Postnatal Depression Scale (EPDS) was used.^15^ EPDS was added at the end of the four questionnaires used in the study.

EPDS is a self-applied scale composed of 10 topics whose options are scored from 0 to 3 (Likert scale) depending on the presence and the intensity of depressive symptoms. EPDS examines an individual's emotional condition in the week before assessment. The questions evaluate depressive and disphoric mood, loss of pleasure, reduced energy in everyday tasks, and intensive thoughts regarding death and suicide. The psychometric validity of the scale is of 60% for positive predictive value to evaluate depression at a cutoff point of ≥13 (59.5% sensitivity, 88.4% specificity). Therefore a score of 13 or less is considered normal, whereas a score higher of 13 has been suggested as an indicator for depression.

### Smoking

To examine the association between smoking status and perinatal depression respondents were categorized as follows:

(1) Nonsmokers: respondents who never smoked in their life or had quit smoking before being aware of their pregnancy (at least 6–12 months before). These women reported to be nonsmoker at all time points during and after pregnancy.
(2) Recent ex-smokers: respondents who had been smoking and made successful quit attempts after being informed of their pregnancy. These women reported not to be smoking at all time during their pregnancy.
(3) Smokers: respondents who reported to be smoking at all time points before and during their pregnancy.

Women who decreased daily cigarette consumption during pregnancy were not considered as quitters (recent ex-smokers), but they were incorporated in the third category of “smokers.” The main reason was that their attempts were not characterized with stability.

## Data Analysis

Statistical analyses were performed with the chi-square test for categorical variables and Student's *t*-test for numeric variables. Binary logistic regression analysis was used to evaluate the association of independent variables with (1) antenatal depression, (2) postnatal depression, and (3) smoking during pregnancy. Significance was set at *p* < 0.05. The statistical software used was SPSS 25.0.

## Results

### Characteristics of respondents

Initially 262 women were approached. Of these 56 declined to participate owing to various reasons that have been documented. Finally, 206 women volunteered and were included. Table 1 gives an overview of the distribution of the demographic variables among respondents. The mean age of respondents was 30.1 years (range, 18–44 years; standard deviation = 7.15 years).

**Table 1.** Characteristics of Respondents

Variables	***n***	%
Nationality		
Greek	157	76.2
Other	49	23.8
Age		
18–25	42	20.4
26–35	90	43.7
36–45	74	35.9
Residence		
Urban	137	66.5
Rural	69	33.5
Marital status^a^		
With intimate relationship	169	82.0
Without intimate relationship	32	15.5
Other	5	2.4
Educational level^b^		
Low	40	19.4
Medium	98	47.6
High	68	33.0
Number of existing children		
None	62	30.1
1	84	40.8
2	23	11.2
3	16	7.8
4	11	5.3
More than 4	10	4.9

^a^ “With intimate relationship” (married or in relationship with the father), “Without intimate relationship”(single, divorced, separated, or widowed), and “Other”(the relationship with the father was complicated).

^b^ “Low” (women who have not obtained a high school degree), “Medium” (women who obtained a high school degree) and “High” (women who had a college or university degree).

### Type of conception

Of the 206 women assessed 156 (75.7%) conceived *in vivo*. A small amount of women (13.1%, *n* = 27) conceived through artificial insemination and 11.2% (*n* = 23) through *in vitro* fertilization.

Among the 156 women who conceived *in vivo*, for 16.7% (*n* = 26) conception was coincidental, 9% (*n* = 14) conceived immediately after trying, 14.7% (*n* = 23) tried from 1 to 6 months, 19.9% (*n* = 31) from 6 to 12 months, and 39.7% (*n* = 62) tried for over a year. Regarding women who conceived through artificial insemination 1.5% (*n* = 3) attempted once, 3.4% (*n* = 7) twice, 4.4% (*n* = 9) three times, and eight women (3.9%) made more than three efforts. Among women who conceived through *in vitro* fertilization 1.9% (*n* = 4) tried once, 1.9% (*n* = 4) twice, 3.4% (*n* = 7) three times, and 3.9% (*n* = 8) made more than three attempts to conceive.

### Parturition and neonate variables

Delivery was performed vaginally for 54.9% (*n* = 113) women and by caesarean section for 45.1% (*n* = 93) women. Among neonates 51.9% (*n* = 107) were females and 48.1% (*n* = 99) males. Most of them (72.3%, *n* = 149) had an appropriate birth weight, whereas 27.7% (*n* = 57) had low birth weight. Thirty-four percent (*n* = 70) of newborns were admitted at the Neonatal Intensive Care Unit (NICU).

### Patterns of smoking before and after pregnancy

In relation to smoking before pregnancy, 28.2% (*n* = 58) of women smoked for over 5 years, 25.2% (*n* = 52) smoked from 1 to 5 years, and 15% (*n* = 31) smoked for about a year. In addition, 31.6% (*n* = 65) of the respondents did not smoke before pregnancy. Regarding the average daily cigarette consumption before pregnancy 13.6% (*n* = 28) of women smoked up to a maximum of five cigarettes per day, 34.5% (*n* = 71) smoked a packet of cigarettes per day, and 20.4% (*n* = 42) smoked more than a pack of cigarettes per day.

Based on answers at the four data collection points respondents were categorized in three smoking patterns regarding smoking during pregnancy: nonsmokers, recent ex-smokers, and smokers. According to data analysis, recent ex-smokers were 29.1% (*n* = 60) and smokers 39.3% (*n* = 81) of the respondents. Regarding the 31.6% (*n* = 65) of women who did not smoke before pregnancy they remained nonsmokers during that period of time. In conclusion, a high proportion of women (39.3%) were smoking during pregnancy. With regard to average daily cigarette consumption during pregnancy, 25.2% (*n* = 52) smoked up to five cigarettes per day, 10.7% (*n* = 22) smoked a pack of cigarettes daily, and 3.4% (*n* = 7) of smokers consumed more than a pack of cigarettes daily.

### Patterns of depression during and after pregnancy

Antenatal depression was detected in 91 women (44.2%), whereas 129 (62.6%) suffered from postnatal depression. Table 2 shows the distribution of variables in correlation with *antenatal depression* following chi-square analysis and Table 3 shows the distribution of variables in correlation with *postnatal depression* following chi square analysis.

**Table 2.** Distribution of Variables in Correlation with ***Antenatal Depression*** Following Chi-Square Analysis

Variables	***n*** (%)	EPDS >13, ***n*** (%)	***p***
Nationality			
Greek	157 (76.2)	71 (45.2)	0.354
Other	49 (23.8)	20 (40.8)	
Age, years			
18–25	42 (20.4)	10 (23.8)	**0.026**
26–35	90 (43.7)	42 (46.6)	
36–45	74 (35.9)	39 (52.7)	
Residence			
Urban	137 (66.5)	58 (42.3)	0.274
Rural	69 (33.5)	33 (47.8)	
Marital status			
With intimate relationship	169 (82.0)	76 (45.0)	0.886
Without intimate relationship	32 (15.5)	13 (40.6)	
Other	5 (2.4)	2 (40.0)	
Educational level			
Low	40 (19.4)	20 (50.0)	0.056
Medium	98 (47.6)	49 (50.0)	
High	68 (33.0)	22 (32.4)	
Number of existing children			
None	62 (30.1)	38 (61.3)	**0.01**
1	84 (40.8)	25 (29.8)	
2	23 (11.2)	7 (30.4)	
3	16 (7.8)	7 (43.8)	
4	11 (5.3)	6 (54.5)	
More than 4	10 (4.9)	8 (80.0)	
Employment before pregnancy			
Yes	155 (75.2)	66 (42.6)	0.260
No	51 (24.8)	25 (49.0)	
Employment during pregnancy			
Yes	121 (58.7)	51 (42.1)	0.289
No	85 (41.3)	40 (47.1)	
Physical exercise before pregnancy			
Yes	123 (59.7)	58 (47.2)	0.183
No	83 (40.3)	33 (39.8)	
Physical exercise during pregnancy			
Yes	72 (35.0)	30 (41.7)	0.351
No	134 (65.0)	61 (45.5)	
Housekeeping before pregnancy			
Alone	172 (83.5)	78 (45.3)	0.704
With help	25 (12.1)	10 (40.0)	
Someone else	9 (4.4)	3 (33.3)	
Housekeeping during pregnancy			
Alone	148 (71.8)	67 (45.3)	0.631
With help	42 (20.4)	16 (38.1)	
Someone else	16 (7.8)	8 (50.0)	
Nursing of elderly before pregnancy			
Yes	42 (20.4)	25 (59.5)	**0.019**
No	164 (79.6)	66 (40.2)	
Nursing of elderly during pregnancy			
Yes	42 (20.4)	25 (59.5)	**0.019**
No	164 (79.6)	66 (40.2)	
Nursing of handicapped before pregnancy			
Yes	9 (4.4)	5 (55.6)	0.356
No	197 (95.6)	86 (43.7)	
Nursing of handicapped before pregnancy			
Yes	9 (4.4)	5 (55.6)	0.356
No	197 (95.6)	86 (43.7)	
Economic stress during pregnancy			
Yes	154 (74.8)	72 (46.8)	0.131
No	52 (25.2)	19 (36.5)	
Type of conception			
*In vivo*	156 (75.7)	56 (35.9)	**0.000**
Artificial insemination	27 (13.1)	17 (63.0)	
*In vitro* fertilization	23 (11.2)	18 (78.3)	
Time interval until *in vivo* conception			
More than 12 months	62 (39.7)	33 (53.2)	**0.000**
6–12 months	31 (19.9)	18 (58.1)	
1–6 months	23 (14.7)	2 (8.7)	
Immediately	14 (9.0)	2 (14.3)	
Coincidentally	26 (16.7)	1 (3.8)	
Attempts of artificial insemination			
None	179 (87.4)	74 (41.0)	**0.012**
1	3 (1.5)	1 (33.3)	
2	7 (3.4)	3 (42.9)	
3	9 (4.4)	6 (66.7)	
More than 3	8 (3.9)	7 (87.5)	
Attempts of *in vitro* fertilization			
None	183 (88.8)	73 (39.9)	**0.01**
1	4 (1.9)	1 (25.0)	
2	4 (1.9)	3 (75.0)	
3	7 (3.4)	6 (85.7)	
More than 3	8 (3.9)	8 (100.0)	
Smoking during pregnancy			
Nonsmokers	65 (31.6)	19 (29.2)	**0.001**
Recent ex-smokers	60 (29.1)	38 (63.3)	
Smokers	81 (39.3)	34 (42.0)	
Average daily cigarette consumption before pregnancy			
Nonsmokers	65 (31.6)	19 (29.2)	**0.027**
Up to 5 cigarettes	28 (13.6)	15 (53.6)	
1 packet	71 (34.5)	34 (47.9)	
More than 1 packet	42 (20.4)	23 (54.8)	
Average daily cigarette consumption during pregnancy			
Nonsmokers	125 (60.7)	57 (45.6)	**0.014**
Up to 5 cigarettes	52 (25.2)	15 (28.8)	
1 packet	22 (10.7)	14 (63.6)	
More than 1 packet	7 (3.4)	5 (71.4)	

Bold values indicate the significant relationship between the socio-demographic variables mentioned and antenatal depression.

EPDS, Edinburgh Postnatal Depression Scale.

**Table 3.** Distribution of Variables in Correlation with ***Postnatal Depression*** Following Chi-Square Analysis

Variables	***n*** (%)	EPDS >13, ***n*** (%)	***p***
Nationality			
Greek	157 (76.2)	104 (66.2)	**0.041**
Other	49 (23.8)	25 (51.0)	
Age, years			
18–25	42 (20.4)	14 (33.3)	**0.012**
26–35	90 (43.7)	62 (68.9)	
36–45	74 (35.9)	53 (71.6)	
Residence			
Urban	137 (66.5)	84 (61.3)	0.348
Rural	69 (33.5)	45 (65.2)	
Marital status			
With intimate relationship	169 (82.0)	102 (60.4)	0.289
Without intimate relationship	32 (15.5)	24 (75.0)	
Other	5 (2.4)	3 (60.0)	
Educational level			
Low	40 (19.4)	32 (80.0)	**0.019**
Medium	98 (47.6)	61 (62.2)	
High	68 (33.0)	36 (52.9)	
Number of existing children			
None	62 (30.1)	44 (71.0)	0.461
1	84 (40.8)	51 (60.7)	
2	23 (11.2)	12 (52.2)	
3	16 (7.8)	8 (50.0)	
4	11 (5.3)	8 (72.7)	
More than 4	10 (4.9)	6 (60.0)	
Employment before pregnancy			
Yes	155 (75.2)	101 (65.2)	0.126
No	51 (24.8)	28 (54.9)	
Employment during pregnancy			
Yes	121 (58.7)	77 (63.6)	0.415
No	85 (41.3)	52 (61.2)	
Physical exercise before pregnancy			
Yes	123 (59.7)	93 (75.6)	**0.000**
No	83 (40.3)	36 (43.4)	
Physical exercise during pregnancy			
Yes	72 (35.0)	46 (63.9)	0.452
No	134 (65.0)	83 (61.9)	
Housekeeping before pregnancy			
Alone	172 (83.5)	111 (64.5)	0.088
With help	25 (12.1)	11 (44.0)	
Someone else	9 (4.4)	7 (77.8)	
Housekeeping before pregnancy			
Alone	148 (71.8)	96 (64.9)	0.121
With help	42 (20.4)	21 (50.0)	
Someone else	16 (7.8)	12 (75.0)	
Nursing elderly before pregnancy			
Yes	42 (20.4)	31 (73.8)	0.065
No	164 (79.6)	98 (59.8)	
Nursing elderly during pregnancy			
Yes	42 (20.4)	31 (73.8)	0.065
No	164 (79.6)	98 (59.8)	
Nursing handicapped before pregnancy			
Yes	9 (4.4)	6 (66.7)	0.549
No	197 (95.6)	123 (62.4)	
Nursing handicapped during pregnancy			
Yes	9 (4.4)	6 (66.7)	0.549
No	197 (95.6)	123 (62.4)	
Economic stress during pregnancy			
Yes	154 (74.8)	102 (66.2)	**0.048**
No	52 (25.2)	27 (51.9)	
Type of conception			
*In vivo*	156 (75.7)	88 (56.4)	**0.02**
Artificial insemination	27 (13.1)	20 (74.1)	
*In vitro* fertilization	23 (11.2)	21 (91.3)	
Time interval until *in vivo* conception			
More than 12 months	62 (39.7)	47 (75.8)	**0.000**
6–12 months	31 (19.9)	22 (71.0)	
1–6 months	23 (14.7)	9 (39.1)	
Immediately	14 (9.0)	6 (42.9)	
Coincidentally	26 (16.7)	4 (15.4)	
Attempts of artificial insemination			
None	179 (86.9)	109 (60.9)	**0.012**
1	3 (1.5)	2 (66.7)	
2	7 (3.4)	5 (71.4)	
3	9 (4.4)	6 (66.7)	
More than 3	8 (3.9)	7 (87.5)	
Attempts of artificial insemination			
None	183 (88.8)	108 (59.0)	**0.04**
1	4 (1.9)	4 (100.0)	
2	4 (1.9)	3 (75.0)	
3	7 (3.4)	6 (85.7)	
More than 3	8 (3.9)	8 (100.0)	
Type of delivery			
Vaginal delivery	113 (54.9)	74 (65.5)	0.214
Caesarean section	93 (45.1)	55 (59.1)	
Gender of neonate			
Male	99 (48.1)	66 (66.7)	0.156
Female	107 (51.9)	63 (58.9)	
Complications during labor			
Yes	27 (13.1)	19 (70.4)	0.251
No	179 (86.9)	110 (61.5)	
Low birth weight			
Yes	57 (27.7)	39 (68.4)	0.184
No	149 (72.3)	90 (60.4)	
Admittance in NICU			
Yes	70 (34.0)	49 (70.0)	0.077
No	136 (66.0)	80 (58.8)	
Smoking during pregnancy			
Nonsmokers	65 (31.6)	27 (41.5)	**0.000**
Recent ex-smokers	60 (29.1)	35 (58.3)	
Smokers	81 (39.3)	67 (82.7)	
Average daily cigarette consumption before pregnancy			
Nonsmokers	65 (31.6)	27 (41.5)	**0.000**
Up to 5 cigarettes	28 (13.6)	20 (71.4)	
1 packet	71 (34.5)	49 (69.0)	
More than 1 packet	42 (20.4)	33 (78.6)	
Average daily cigarette consumption during pregnancy			
Nonsmokers	125 (60.7)	62 (49.6)	**0.000**
Up to 5 cigarettes	52 (25.2)	42 (80.8)	
1 packet	22 (10.7)	18 (81.8)	
More than one packet	7 (3.4)	7 (100.0)	

Bold values indicate the significant relationship between the socio-demographic variables mentioned and postnatal depression.

NICU, Neonatal Intensive Care Unit.

Binary logistic regression regarding *antenatal depression* showed interesting results. Association between smoking before and during pregnancy and expression of depressive symptoms was significant (*B* = 0.568; *p* = 0.026 and *B* = 1.238; *p* = 0.009, respectively). Average daily cigarette consumption before and during pregnancy was also significantly correlated (*B* = 1.110; *p* = 0.001 and *B* = 1.167; *p* = 0.002, respectively).

Women who experienced economic stress during pregnancy reported significantly more depressive symptoms (*B* = 1.092; *p* = 0.022), whereas attempts of artificial insemination and *in vitro* fertilization increased the risk of depression (*B* = 0.905; *p* = 0.011 and *B* = 1.1733; *p* = 0.004). Finally knowledge of risks of tobacco consumption during pregnancy tended to correlate with antenatal depression (*B* = 0.551; *p* = 0.058 > α = 0.05).

Furthermore, binary logistic regression concerning *postnatal depression* showed significance between smoking during pregnancy and experience of symptoms (*B* = 1.757; *p* = 0.005). A statistically significant association was also observed between average daily consumption of cigarettes during pregnancy and depression (*B* = 1.402; *p* = 0.002). Low birth weight and potential necessity of admission in the NICU increased the risk of postnatal depression (*B* = 2.572; *p* = 0.003 and *B* = 2.180; *p* = 0.005, respectively). Maternal age was associated with a significantly higher risk of depression (*B* = 0.697; *p* = 0.004). Nursing of elderly before and during pregnancy were also significantly correlated (*B* = 1.490; *p* = 0.018 and *B* = 1.490; *p* = 0.018).

Experience of antenatal depression was significantly associated with postnatal depression as well (*B* = 1.341; *p* = 0.004). On the contrary, physical exercise before pregnancy (*B* = −1.238; *p* = 0.011) and a higher educational level (*B* = −578; *p* = 0.048) were negatively correlated with the expression of depressive symptoms. Smoking and average daily cigarette consumption before pregnancy were not significant determinants of postnatal depression, whereas knowledge of risks concerning smoking during pregnancy tended to associate with postnatal depression (*B* = 0.566; *p* = 0.054).

Binary logistic regression was also used to evaluate which variables determine smoking during pregnancy. Smoking and average daily cigarette consumption before pregnancy were significant determinants (*B* = 0.568; *p* = 0.026 and *B* = 1.465; *p* = 0.025, respectively). Stress experienced during pregnancy owing to economic factors was also significantly associated (*B* = 1.110; *p* = 0.001). Older women were more likely to continue smoking during pregnancy (*B* = 0.061; *p* = 0.021). Physical exercise before and during pregnancy were negatively correlated with smoking (*B* = −1.831; *p* = 0.000 and *B* = −1.583; *p* = 0.000).

In addition, the time interval until conception as well as the number of attempts of artificial insemination and *in vitro* fertilization enhanced significantly the potential of quitting smoking during pregnancy (*B* = −1.422; *p* = 0.001, *B* = −1.475; *p* = 0.019 and *B* = −1.110; *p* = 0.001). Knowledge of the risks of smoking along with the persistent and detailed consultation from the obstetrician significantly reduced smoking during pregnancy (*B* = −1.110; *p* = 0.001 and *B* = −1.238; *p* = 0.009).

The follow-up investigation revealed that 40 (66.6%) women of the group that ceased smoking during pregnancy (recent ex-smokers) have returned to their smoking habits. Regarding the time interval, 12 (20%) restarted smoking almost 6 months after labor, whereas the majority of this group (28, 46.6%) revealed recommencing smoking a year after giving birth.

## Discussion

The aim of this study was mainly to obtain a clear insight into the possible association between smoking patterns and perinatal depression, taking into consideration a variety of sociodemographic variables. Furthermore, it was attempted to evaluate the reasons that prevent women from quitting smoking during pregnancy. The strength of this study is its longitudinal design as respondents were questioned four times at four different perinatal periods and furthermore a follow-up investigation took place 2 years after labor. Therefore, it was possible to determine mood fluctuations and potential variations in smoking patterns both before childbirth and after pregnancy.

During the past decades the percentage of female smokers increased and tends to reach especially high levels. Despite the mounting evidence about negative impact of smoking on fertility and pregnancy, a significant amount of women fail to quit smoking even after conception.^3,14,16^

After the confirmation of pregnancy (and under the burden of responsibilities toward their growing child), 29.1% of women abstained from smoking, whereas 39.3% continued smoking although they were aware of its negative consequences on the child's health. Our results showed that the larger amount of smokers during pregnancy reduced tobacco exposure to minimize potential risks for the fetus, a finding that was also confirmed in previous studies.^11,17,18^

Binary logistic regression analysis, as well as chi-square analysis (as given in Tables 2 and 3), revealed the relative strength of association of all investigated independent variables with antenatal and postnatal depression as well as with smoking during pregnancy.

A significant *relationship* between smoking and average daily cigarette consumption before and during pregnancy and the expression of antenatal depressive symptoms was found. Recent studies including women who continued smoking and women who spontaneously quitted after learning about their pregnancy showed that smokers reported significantly less antenatal depression symptoms than spontaneous quitters.^19–21^

These results suggest the magnitude of difficulty for pregnant smokers to quit this habit, as it is associated with emotional disorders present throughout pregnancy. Quitting smoking is a difficult task that overwhelms each individual as it is actually rehabilitation from a highly addictive substance, such as nicotine. Consequently, it is accompanied with withdrawal symptoms, mood changes, and depressive emotions. During pregnancy women are very vulnerable and the difficulty of quitting smoking is an extra burden.

Furthermore, economic stress during pregnancy and increased attempts of artificial insemination and *in vitro* fertilization tended to correlate with antenatal depression. Previous studies also indicated the association among these risk factors and antenatal depression.^3,22,23^

Postnatal depression binary logistic regression analysis showed positive correlation between smoking during pregnancy and average daily tobacco consumption with the expression of depressive symptoms. Similar studies also indicated the decrease of postnatal depression among women who managed to quit smoking during pregnancy.^5,18,21^ This reduction is likely owing to the time interval between the onset of quitting efforts and postnatal period and therefore withdrawal symptoms and depressive behavior were slightly minimized.

Low birth weight and admission in the NICU were also positively related to the risk of postnatal depression. These results agree with recent studies where the arrival of a healthy offspring was mentioned to spark a new circle of joyful emotions accompanied by optimism.^17,24^

Basically, smokers after birth acknowledge the consequences of smoking during pregnancy on their offspring. In any case, they are overwhelmed with emotions of guilt, which can be aggravated depending on the severity of infant morbidity. Some studies showed high level of postnatal depression in this category of women, which was attributed to the emotional tension they experience.^6,25^ This emphasizes the fact that both smoking cessation and potential hazards should be well addressed during prenatal consultations.

Maternal age was associated with a significantly higher risk of depression as was also suggested by previous studies.^4,5^ Furthermore, our study showed that nursing of elderly people before and during pregnancy burdened emotionally women leading to increased levels of depression.

Experience of antenatal depression was also associated with postnatal depression symptoms. There is a clear relationship among antenatal and postnatal depression as recently suggested.^2,3,9,23^

On the contrary, physical exercise before pregnancy and higher educational level inhibited significantly the expression of depressive symptoms. The preventive function of exercise was shown in previous studies.^3,4,22^

Binary logistic regression was also used to determine statistical significance between multiple variables and smoking during pregnancy. The inability of quitting smoking during pregnancy seemed to be significantly related to smoking behavior and average daily cigarette consumption before pregnancy. Stress experienced during pregnancy owing to economic factors also correlated negatively to the attempts of quitting.^2,4,23^

Older women were more likely to continue smoking during pregnancy, whereas physical exercise before and during pregnancy were significant contributors in abstaining from smoking.^13,18^

In addition, the increase in time interval until conception as well as in efforts of artificial insemination and *in vitro* fertilization enhanced significantly the potential of quitting smoking during pregnancy. Adequate knowledge of potential risks for the fetus along with detailed notification from the obstetrician were significant in the attempt of quitting. On the contrary, economic stress during pregnancy and related low socioeconomic life status can lead to unsuccessful quitting. These results agree with recent studies implying that chronic smokers encounter severe difficulties in quitting smoking during pregnancy.^5,21^

Women who ceased smoking during pregnancy are most likely to recommence this habit shortly after giving birth. The period after pregnancy is regarded as a period full of anxiety and stress. The modification of everyday life routine burdens emotionally these women and restarting smoking after childbirth seems to act as a way to capture fragments of their previous life, offering feelings of relaxation and pleasure. Failing to retain abstaining from smoking during the period after pregnancy is also described in recent studies, revealing an elevated amount of women returning to their previous smoking habits.^14,19,21^

Given the clear dose–response relationship between smoking patterns during pregnancy and the presence of perinatal depression established in this study, it is suggested that obstetricians should take enough time to examine not only obstetric parameters but also the life status of the pregnant women. Smoking cessation must be a priority and to achieve this goal it is important that health professionals involved in the care of pregnant women provide to these patients more than a mere clarification of the risks associated with smoking.

Detection and treatment of perinatal depression and smoking cessation advice can prevent the appearance of more severe health problems in both new mothers and newborns. These women need more specialized care and a strategy pattern must be promoted to cease smoking. Counseling must be tailored to their special needs and attention must be paid to the methodology used, for example, using appropriate language and even continuous psychological treatment.

## Conclusion

In conclusion, mental health of women during the period before and after childbirth must be protected with special attention to avoid potential hazards. Smoking patterns were associated with both antenatal and postnatal depression. Owing to the emotional particularity of women during pregnancy mindful assessment and intervention for the cessation of smoking should be considered to support the health of new mothers.

## Abbreviations Used

EPDS: Edinburgh Postnatal Depression Scale
NICU: Neonatal Intensive Care Unit
